# ‘Is it me or my illness?’: self-illness ambiguity as a useful conceptual lens for psychiatry

**DOI:** 10.1192/bjp.2025.10367

**Published:** 2026-03

**Authors:** Roy Dings, Anna Golova

**Affiliations:** Faculty of Philosophy, Theology and Religious Studies, Radboud University, Nijmegen, The Netherlands; Department of Psychiatry, University Medical Center Utrecht, Utrecht, The Netherlands; Lemon Tree Interdisciplinary Center for Psychiatry, Psychotherapy and Philosophy, Amsterdam University Medical Center, Amsterdam, The Netherlands; Faculty of Philosophy, University of Oxford, Oxford, UK; Uehiro Oxford Institute, University of Oxford, Oxford, UK; Merton College, University of Oxford, Oxford, UK

**Keywords:** Philosophy, ethics, patients, psychotherapy, diagnosis and classification

## Abstract

The complex relation between a person and their mental disorder is a recurring theme in (reflections on) psychiatric practice. As there is no uncontested concept of ‘self’, nor of ‘mental disorder’, the ‘self-illness’ relation is riddled with ambiguity. In this feature article, we summarise recent philosophical work on the phenomenon of ‘self-illness ambiguity’, to provide conceptual tools for psychiatric reflections on the self-illness relation. Specifically, we argue that the concept of self-illness ambiguity may contribute to patients’ self-understanding and shed light on how paradigms of care and research should be revised in order to help clinicians support that self-understanding. We also suggest that the concept of self-illness ambiguity may improve the understanding of particular mental disorders, and may offer conceptual tools to address various ethical matters (including stigma and responsibility).

In the early 1990s, sociologist David A. Karp studied self-help groups for affective disorders and found that many attendants of these groups struggled with the complexity of their illness and its relation to themselves and their actions. Karp^1^ described several examples of this phenomenon, which he called ‘illness ambiguity’:

‘One woman whose husband was a manic depressive said that sometimes “it is unclear whether the illness is talking or whether [he] is talking”. In a different meeting, a woman, who described a history of beginning to take college courses only to drop out, offered the following analysis of her behavior: “I would always start courses and then drop out. I used to think I was easily bored. Now I don’t blame myself or the class. It’s not me or the class, it’s the illness”. Yet another woman who was undecided about returning to work as a preschool teacher wondered out loud: “Do I not want to go back to it, or is it because of the illness that I don’t want to return?”. [Another participant added:] “I’m trying to discover my basic personality aside from the disease. That’s why I’m at these meetings, to discover which is which”.’^1^

Psychiatrist and philosopher John Z. Sadler later picked up on the same phenomenon when he discussed ‘self-illness ambiguity’ as a ‘fundamental blurring of mental illness and the personal self’.^2^ More than the term ‘illness ambiguity’, the term ‘self-illness ambiguity’ highlights that this is a relational phenomenon, involving various relations between selfhood and mental disorder.^a^ However, elucidating how self and mental illness relate requires us to have clear concepts of both selfhood and mental disorder.^3^ That is, one’s view of the relation between self and mental illness (hereinafter, ‘self-illness relation’) will depend on both how one construes mental disorders and how one construes selfhood. Importantly, the fact that there is no uncontested concept of mental disorder, nor of selfhood, adds to the complexity of the self-illness relation. It also means that there is a lot of ambiguity that people with mental disorders can experience and that can hinder them in attempts at making sense of their relation to their illness, and its impact on their agency.

The complexity of the self-illness relation is a recurring theme in (reflections on) psychiatry, including in the *British Journal of Psychiatry* (see, e.g. ^4,5^). We wish to flag up that there has also been considerable philosophical work on this topic in recent years, focusing on the phenomenon and concept of self-illness ambiguity as a ‘difficulty in distinguishing one’s self, or “who one is”, from a mental disorder or diagnosis’^6^ (see also ^7–22^).^b^ In this feature article, we summarise this recent and ongoing conceptual work on self-illness ambiguity and discuss some important connections of these philosophical ideas to psychiatric practice. In particular we show, in the following section, how the concept of self-illness ambiguity may contribute to supporting patients’ self-understanding in clinical practice. Then, in ‘Self-illness ambiguity as a useful conceptual lens for policy, ethics and research in psychiatry’, we explore how self-illness ambiguity may be a useful conceptual lens through which to address broader matters in psychiatric research, policy and ethics. Overall, this feature article aims to enable more clinical and empirical research on self-illness ambiguity, alongside the evolving conceptual work in philosophy.

## Self-illness ambiguity as a useful conceptual lens for clinical practice: facilitating patients’ self-understanding

In this section, we discuss three key distinctions within the concept of self-illness ambiguity (its relations, modes and perspectives) and show how they may be beneficial to clinical practice.^c^ Specifically, we illustrate how these may be used by a clinician in assisting a hypothetical patient (‘Peter’), diagnosed with autism spectrum disorder, in his quest for self-understanding.^d^ The three distinctions (and their interconnectedness) are summarised in Table 1.

**Table 1:** Relations, modes and perspectives of self-illness ambiguity

Relatum	Mode	Perspective	Illustration	
Identity	Reflective	1PP	‘How do I relate to my illness?’	
		3PP	Token:	‘How does person B relate to their illness?’
			Type:	‘How does selfhood relate to psychopathology?’
	Unreflective	1PP	Feeling ambiguous about oneself in relation to one’s illness	
Agency	Reflective	1PP	‘Is it me or my illness that makes me do/think/feel this?’	
		3PP	Token:	‘Is it B or their illness that makes them do/think/feel this?’
			Type:	‘How does agency relate to psychopathology?’
	Unreflective	1PP	Feeling ambiguous about an action/thought/feeling	

1PP, first-person perspective; 3PP, third-person perspective.

First, it may be helpful to specify the exact ‘relation’ that is at stake within self-illness ambiguity. On the one hand, we may investigate identity: how does a mental disorder relate to myself, i.e. ‘who I am’?^3^ On the other hand, we may investigate agency, e.g. should an aspect of my agency, such as an action, thought, feeling, desire or inclination, be attributed to myself or to my disorder? (see also ^17^). The agency form of self-illness ambiguity is central to the common ‘slogan’ of ‘is it me or my illness that is making me do this?’. Clearly, agency and identity are interrelated (e.g. one’s construal of one’s identity may affect one’s agency, and one’s actions may in turn feed into one’s identity), but it may nevertheless be useful for clinical practice to distinguish them conceptually.

To illustrate, when a patient utters an agency-related worry, the therapist may strategically (for therapeutic purposes) shift towards talking about identity. For instance, suppose our hypothetical patient Peter asks whether it is ‘his autism’ that makes him behave in a certain way. The therapist may respond to Peter by asking whether and to what extent Peter thinks that this question stems from how he has construed his relationship to his diagnosis. It may be helpful to both Peter and his therapist to explicate possibly implicit conceptualisations of the condition. For instance, it might turn out that Peter adopts a reified conceptualisation – that is, he thinks of autism as a ‘thing’ to relate to (at the identity level) and a thing that ‘makes’ him act in certain ways (at the agency level).^6^

This distinction between identity and agency may also be useful for clinical practice, because questions regarding identity might require a different response in a therapeutic context than those regarding agency. For instance, an agency-related utterance of ‘is it my autism that is making me behave this way?’ could signal that Peter worries about matters of responsibility, which is something the therapist may tap into.

Here, it is also important for clinicians to acknowledge that, in the process of self-understanding, patients may go back and forth in how they relate to their condition or may relate differently to different aspects of it^23^ (see also ^4–6,22^). Even if someone thinks of ‘their illness’ in a negative manner, they may still construe certain elements of it as positive.^2^ Clinicians are asked to be sensitive to such nuances, including, for instance, to the ways in which distancing oneself from one’s condition as a whole, or from (the responsibility for) certain actions, may be harmful for some patients^1,21,24^ and helpful for others.^23^ Indeed, some philosophers have argued that ambiguity itself may offer insights and resources for recovery^14,16,22^ (see ‘Self-illness ambiguity as a useful conceptual lens for policy, ethics and research in psychiatry’, below).

As a second conceptual distinction in self-illness ambiguity, we may discern the specific ‘mode’ in which self-illness ambiguity is encountered. On the one hand, one may address identity or agency in a reflective mode, where one consciously deliberates about the ambiguity and employs conceptual tools to establish clarity. One might do this while sitting in an armchair at home, or while having a discussion with one’s partner or therapist. On the other hand, one may be confronted with these ambiguous relations in an unreflective mode when going about one’s day-to-day affairs. In other words, whenever people are not consciously deliberating (i.e. most of daily life), they may still experience their self-illness relation as ambiguous.*^6^*

Distinguishing these two modes is important because it may help to better understand the processes underlying self-illness ambiguity. According to Dings and Glas,^6^ self-illness ambiguity is strongly linked to the interplay of these two modes. In a nutshell, they suggest that ambiguity emerges due to a mismatch or incoherence between how people experience themselves (in an unreflective mode) and how they deliberately understand themselves (in a reflective mode).

To illustrate, suppose that Peter has been informed that autism is a neurodevelopmental disorder. This conceptualisation feeds into the ‘reflective’ level, where Peter constructs a self-narrative of someone who ‘has’ autism. However, it may be that this conceptualisation does not feel right to Peter in his day-to-day life. The pervasiveness and omnipresence of (some of his) symptoms make him feel like he ‘is autistic’ rather than ‘has autism’. Furthermore, some of his putative symptoms (e.g. his strong and highly focused interests) feel as truly ‘his’ rather than the result of an abnormal neurodevelopmental trajectory. Thus, there is incoherence between how Peter thinks about himself and how he experiences himself, giving rise to ambiguity. In such a case, to reduce ambiguity the clinician may, for example, work with Peter on revising his self-narrative to find a conceptualisation of autism that ‘fits’ his self-experience, or propose body-focused therapies to further explore Peter’s bodily and affective feelings with respect to his diagnosis.^6^

A third conceptual distinction is the ‘perspective’ from which one faces or considers self-illness ambiguity (first- or third-personal^10^), involving both particular token- and general-type angles (the latter of which we will consider in ‘Self-illness ambiguity as a useful conceptual lens for policy, ethics and research in psychiatry’, below). Taking the token angle here means asking how a particular individual relates to a particular illness, which seems especially relevant to clinical practice. On the one hand, this may occur from a first-person perspective (1PP) (e.g. at the agency level, someone struggles to determine whether their lack of appetite is theirs or a symptom of an eating disorder that is in remission). On the other hand, such questions may arise from a third-person perspective (3PP) (e.g. the patient’s family, their GP or psychiatrist struggles to determine whether the patient’s lack of appetite should be attributed to an eating disorder^25^).

Or consider, again, the hypothetical case of Peter, who struggled with the construal of autism as something he ‘has’. It might be that this construal was provided by his clinician when they emphasised the importance of person-first language. Sadler^2^ previously noted that the phenomenon of self-illness ambiguity puts pressure on the American Psychiatric Association’s directive to use person-first language. Indeed, research on linguistic preferences regarding autism (e.g. ‘being autistic’ versus ‘having autism’) consistently shows that different parties (diagnosed individuals, loved ones and professionals) may have different preferences and that these are often based on their differing conceptions of the self-illness relation (e.g. ^26^).

The distinction between 1PP and 3PP self-illness ambiguity provides a language for different parties to communicate and pinpoint disagreements, including disagreements between patient (1PP) and clinician or family members (3PP) on what to count as symptoms. As Sadler^27^ remarked, ‘[q]uite often with mental illness, what clinicians consider the symptoms and signs of illness may be, for the patient, prized aspects of the personal self’. The process of explicating and addressing disagreements among different perspectives will require the clinician to be open to exploring jointly with the patient how they view their behaviours, thoughts and feelings, and to be willing to revise professional third-personal assessment in light of the patient’s first-person perspective. We believe that clinicians’ sensitivity to matters of self-illness ambiguity will thereby probably strengthen the therapeutic alliance and increase treatment adherence.

Taking these distinctions (regarding relations, modes and perspectives) into account, we can see that self-illness ambiguity covers a variety of related, yet subtly different, phenomena. Indeed, closely re-reading the excerpt from Karp that we started with, while keeping the outlined distinctions in mind, reveals that Karp describes examples of these several slightly different phenomena.

## Self-illness ambiguity as a useful conceptual lens for policy, ethics and research in psychiatry

Where the previous section investigated the usefulness of the concept of self-illness ambiguity for patients’ self-understanding and patient–clinician interaction, the current section explores how self-illness ambiguity may be useful to psychiatric practice in a broader sense, i.e. including research, policy and ethical matters. Whereas in clinical practice, self-illness ambiguity primarily concerns the token level of how particular patients relate to their illness, in these broader domains, self-illness ambiguity pertains to the type level, where the question is how selfhood and psychopathology relate to each other in general: e.g. how should we think about ‘the person’ in relation to schizophrenia?^24^ Nonetheless, as will become apparent below, such general-type questions do, of course, interact with token questions about particular cases in clinical contexts.^e^

The third-personal type angle is commonly adopted by philosophers or scientists. For psychiatric research, conceptual work on self-illness ambiguity may offer new insights into particular mental disorders, e.g. their emergence, the consequences of diagnostic labels, illness phenomenology and treatment. For example, McConnell and Golova^16^ argue that recovery from addiction may require going through a period of ambiguity about one’s established addiction self-narrative in order to make room for positive self-transformation (see also ^18^). Drożdżowicz^14^ shows that self-illness ambiguity may similarly positively affect processes of recovery from anorexia nervosa, because the various tensions in experiences of self-illness ambiguity may serve as cues for reflection and resources for self-understanding. Bortolan^11^ shows how, in severe anxiety, alterations in so-called ‘existential feelings’ (roughly, affective background orientations on the world) mediate the interplay between the two modes (reflective and unreflective) of self-illness ambiguity (see also ^19^). Carls-Diamante^13^ addresses self-illness ambiguity in bipolar disorder and provides a taxonomy of self-illness relations in a reflective mode (see also ^17^). Dings et al^9^ examine how self-illness ambiguity in personality disorders may result in the particularly complex phenomenon of ‘self–self ambiguity’, in which there is no longer a solid point from which to engage in clarificatory endeavours of self-understanding.

Taking such philosophical work into account, paradigms in psychiatric research, as well as in care and policy, should be sensitive to the complexity of the self-illness relation and potential ambiguities therein. We wish to highlight that many of the existing paradigms in psychiatry seem to presuppose a clear (non-ambiguous) stance on the self-illness relation, and seldom leave room for the possibility of ambiguity.^2,22^ For instance, research and treatment paradigms rely on seemingly dichotomous or binary concepts such as illness- and self-insight, ego-dystonicity and -syntonicity, self-concept clarity and identity diffusion.

Relating this back to clinical contexts, the conceptual space that is opened up by the notion of self-illness ambiguity can be beneficial, e.g. since dichotomous concepts may make clinicians or patients feel urged to ‘pick a side’ and attribute behaviours or feelings to either a mental disorder or the person, even if they have a sense that neither option is fully sufficient. Consider, in this regard, our hypothetical example of Peter, who embraced some putative symptoms (e.g. his strong and highly focused interests) as his own, but not others (e.g. sensory overload), or felt unsure about yet other symptoms that he experienced as ‘in-between’ his and not his. This escapes potential dichotomous interpretations of concepts such as ego-syntonicity or illness-insight, even more so because, as highlighted above, clinicians, researchers, patients and loved ones may all have different perspectives on the self-illness relation. As noted by James Marcia,

‘The histrionic person may insist that their emotional storms are simply “being honest”. The obsessive–compulsive individual can prize “being cautious, thoughtful, and consistent”. The schizoid personality can take refuge in valuing “solitude, creativity, and introspectiveness”.’^28^

At the level of care policy, paradigms of ‘self-management’ and ‘person-centred care’ in psychiatry may likewise be problematised given the ambiguity inherent in the self-illness relation.^6,29,30^ Self-illness ambiguity also seems particularly relevant to self-understanding in the context of a recovery paradigm, in which ‘defining the self apart from the illness, the illness as only a part of the self’^31^ is seen as a core component. Again, this recovery framework, as well as much research on self-understanding in the psychiatric context (e.g. on illness-identity and -perception^3^), tends to presuppose that self-illness ambiguity is addressed or even resolved.

This presupposition overlooks the complexity of the process of self-understanding, and may make research susceptible to several simplifications. Based on the distinctions we made in ’Self-illness ambiguity as a useful conceptual lens for clinical practice: facilitating patients’ self-understanding’, above, we can identify several pitfalls to be avoided in research: (a) reification, i.e. a flawed conceptualisation of mental disorders as entities or ‘things’;^6^ (b) a dichotomous construal where a mental disorder either is or is not part of oneself, thereby not allowing for ambiguity;^5,6,15^ (c) an overemphasis on the reflective capacities of agents, thereby ignoring the influence of unreflective agency;^6,17^ and (d) an exclusive focus on global individual identity development, which disregards struggles with one’s agency in particular situations.^7^

A final group of wider benefits of employing the self-illness ambiguity concept relates to important ethical matters. A better understanding of the (complexity of the) self-illness relation seems vital for ethical reasons, for instance, because it feeds into questions about societal stigma and patients’ (sense of) agency, autonomy and responsibility (see, e.g. ^1,2,5,26,32^). As previously mentioned, different people may hold different linguistic preferences regarding the self-illness relation. Not allowing for expression of the full breadth of self-illness relations and their ambiguous elements may be considered epistemically unjust.^5^ Beyond this, self-illness ambiguity has ethical significance whenever patients, their loved ones or clinicians attempt to negotiate how autonomy or responsibility for certain actions relates to people’s diagnoses (e.g. Peter’s relatives may wonder to what extent he can help behaving the way he does). A particularly far-reaching ethical implication concerns clinical contexts in which clinicians may wonder whether a patient’s treatment decision might be ‘their illness talking’ (see ^2,4,8^). Here, too, the complexity and ambiguity of the self-illness relation need to be considered with utmost care.

In conclusion, the complexity of the self-illness relation is a recurring theme in (reflections on) psychiatric practice.^4,5^ Grasping this complexity requires us to address the ambiguity that is often inherent in the self-illness relation.^2,6^ This article has sought to give an overview of recent and ongoing philosophical work on self-illness ambiguity, thereby providing conceptual tools to feed into psychiatric reflections on the complexity of the self-illness relation. Of course, there is some ‘division of labour’, such that philosophers are concerned with conceptual considerations and clinicians are concerned with clinical implications. Nevertheless, as philosophers, we hope to hereby contribute to a fruitful dialogue between the disciplines^2,22^ and to facilitate more clinical and empirical research alongside, and in response to, the evolving conceptual work on self-illness ambiguity.

## Footnotes

a. For simplicity, we here speak generally of ‘the self-illness relation’, but as will become clear below, self-illness ambiguity can pertain to various relations between mental disorder, selfhood, and agency.

b. There are many aspects a person may struggle to relate to and thus it has been argued that self-illness ambiguity is a specific form of the more general phenomenon ‘self-ambiguity’^7,10^ (see also ^20^). Some self-ambiguities relate to the domain of psychiatry and may intersect with self-illness ambiguity (e.g. ‘self-medication ambiguity’^8^ or ‘self-implant ambiguity’^12^), whereas others do not (e.g. ‘self-hunger ambiguity’ or ‘self-upbringing ambiguity’^10^). Here, we focus on self-illness ambiguity, i.e. how people strive to relate to mental disorders.

c. For another recent proposal of distinctions relevant to clinical practice, see ^22^.

d. We acknowledge that many people consider autism to be a neurodevelopmental condition rather than a mental illness. We use it here as an example of a diagnosis that nonetheless falls within the remit of support by psychiatrists and that especially frequently brings up questions about how diagnoses relate to people’s identities and different ways of being.

e. Throughout this feature article, we seek to disentangle matters which are in reality highly intertwined and dynamic. Thus, we discuss research, policy and ethics separately from clinical practice, yet we acknowledge that research and policy issues feed into that practice and, conversely, that clinical practice gives rise to ethical questions.
